# Comparative Study of Three Biological Control Agents and Two Conventional Fungicides against Coriander Damping-off and Root Rot Caused by *Rhizoctonia solani*

**DOI:** 10.3390/plants12081694

**Published:** 2023-04-18

**Authors:** Abdelrazek S. Abdelrhim, Yasmin M. R. Abdellatif, Mohammad A. Hossain, Saud Alamri, Mohammad Pessarakli, Amna M. N. Lessy, Mona F. A. Dawood

**Affiliations:** 1 Department of Plant Pathology, Minia University, Minia 85721, Egypt; 2 Department of Agricultural Botany, Faculty of Agriculture, Ain Shams University, Cairo 11566, Egypt; 3 Department of Genetics and Plant Breeding, Bangladesh Agricultural University, Mymensingh 2202, Bangladesh; 4 Department of Botany and Microbiology, College of Science, King Saud University, Riyadh 11451, Saudi Arabia; 5 School of Plant Sciences, The University of Arizona, Tucson, AZ 85721, USA; 6 Botany and Microbiology Department, Faculty of Science, Assiut University, Assiut 71516, Egypt

**Keywords:** biocontrol agents, conventional fungicides, coriander, damping-off, *Rhizoctonia solani*, root rot

## Abstract

The in vitro and in vivo efficacy of three biocontrol agents, *Trichoderma viride*, *Pseudomonas fluorescence*, and *Bacillus subtilis*, were tested against *Rhizoctonia solani* (AG-4) infection compared to two conventional fungicides (Rizolex-T 50%wettable powder and Amistar 25%). Antifungal enzyme activity was assayed in the culture filtrate of the biocontrol agents. The impact of the tested biocontrol agents on the induction of the coriander immune system was investigated against *R. solani* by assessing the resistance-related enzymes and compounds in biocontrol agent-treated plants compared with the control. The obtained results revealed that all tested biocontrol agents significantly reduced the linear growth of *R. solani,* and *T. viride* recorded the highest inhibition percentage. This could be linked to the ability of *T. viride* to produce higher activities of antimicrobial enzymes, i.e., cellulase, chitinase, and protease, compared to *P. fluorescence* and *B. subtilis*. Applying the tested biocontrol agents significantly alleviated pre- and post-emergence damping-off and root rot/wilt diseases of infected coriander compared with untreated plants. The tested biocontrol agents exhibited significantly higher germination percentage and vigor index of the coriander than the tested fungicides. The tested biocontrol agents significantly minimized the reduction of photosynthetic pigments induced by *R. solani*. In addition, the results showed a significant increase in enzymes/molecules (i.e., phenylalanine, catalase, peroxidase, catalase, superoxide dismutase, phenylalanine ammonia-lyase, phenolics, ascorbic acids, and salicylic acid) involved directly and indirectly in coriander resistance to *R. solani*. The principal component analysis of the recorded data recommended the role of the high accumulation of oxidative parameters (hydrogen peroxide and lipid peroxidation) and the inhibition of phenolic compounds in the downregulation of coriander resistance against *R. solani*. The heatmap analysis results revealed that biocontrol agents, especially *Trichoderma*, enhanced the resistance against *R. solani* via the stimulation of salicylic acid, phenolics, and antioxidant enzymes. Overall, the data recommended the efficacy of biocontrol agents, especially *T. viride*, against *R. solani* infecting coriander plants, which could be an efficient and a safer alternative to conventional fungicides.

## 1. Introduction

Coriander (*Coriandrum sativum* L.) also known as cilantro is an ancient medicinal and aromatic plant and seed spice belonging to the family Apiaceae (Umbelliferae). It is native to the Mediterranean Basin region [1]. Its fruits and leaves are widely used as essential herbal ingredients [2]. In addition to its culinary value, coriander is recognized for its broad spectrum of therapeutic benefits against gastrointestinal problems such as dyspepsia, flatulence, diarrhea, griping discomfort, vomiting flatulent colic, etc. In addition, coriander possesses antiedema, anti-inflammatory, antiseptic, emmenagogue, antihypertensive, lipolytic, myorelaxant, antirheumatic, antineuralgic, and nerve-soothing properties [3,4].

Coriander cultivation is severely affected by several diseases that are major issues affecting its growth, development, and yield. Among these diseases, seedling damping-off, root rot, stem rot, and wilt are considered some of the most damaging ones affecting coriander plants [5]. *Rhizoctonia solani* Kuhn (*Thantephorus cucumeris*) is considered one of the critical soil-borne pathogens worldwide. It causes damping-off incidence of approximately 20% of commercial coriander in coastal California [6]. *R. solani* AG-4 was reported as a crown and root rot causal agent on cilantro in California by Koike et al. [7]. In Egypt, *R. solani* attacks coriander during the growing season and causes severe symptoms of damping-off, root rot, and wilt, which results in significant yield losses [8,9].

Various management strategies were used to control damping-off, wilt, and root rot diseases, including agriculture practices, breeding programs, biological control, and broad-spectrum fungicides. Although using fungicides in controlling damping-off and wilt/root rot diseases could be more effective than other strategies, it establishes an imbalance in the microbial community, rendering it unfavorable for beneficial organisms’ activity [10]. Studies by Balba [11] and Gisi et al. [12] reported that Amistar, with Azoxystrobin as an active ingredient, was registered to manage *R. solani* via inhibition of ATP production of the targeted pathogen by binding to the quinone oxidizing site of cytochrome bc1 complex I and restricting the transfer of electrons from cytochrome b to cytochrome c. Despite the efficacy of Amistar against fungal infection, this fungicide relies on a single-site mode of action that could result in a resistant population when repeatedly applied in the field. On the other hand, the effectiveness of Rizolex-T 50% varied among anastomosis groups of *R. solani*. Its mode of action depends on inhibiting the phospholipids’ biosynthesis, leading to the inhibition of fungal growth [13]. However, environmental pollution and its negative effect on mammals and other beneficial living organisms due to the extensive use of one or both fungicides could result in the development of a resistant population that was reported to occur in other plant pathogen populations. Thus, comparative studies using biological controlling agents versus conventional fungicides are needed to select the best microorganism able to suppress the disease severity and determine its potentiality.

Biological control is considered one of the most effective, safe, and useful for the environment [14,15]. The most known and effective biocontrol agents that are tremendously used in controlling plant diseases are *Trichoderma viride*, *Pseudomonas fluorescence*, and *Bacillus subtilis*. In this regard, it has been reported that *Trichoderma* spp. significantly regulates plant growth and suppresses plant pathogenic microorganisms [16,17,18]. Various plant diseases, especially those of the root such as damping-off, root rot, and wilt diseases, were successfully controlled using *Trichoderma* spp. [19,20,21].

Trichoderma isolates colonized plant roots and induces plant resistance to different biotic and abiotic stresses [22]. Various mechanisms were reported explaining how Trichoderma suppresses plant diseases. It directly affects the pathogen by overgrowth and secreting toxic metabolites, including antimicrobial enzymes and compounds [23,24]. A study by Van Wees et al. [25] reported two signaling pathways involving jasmonate, salicylic acid, and ethylene resulted in a molecular-based interaction between the Trichoderma and plant: the first, mediated by salicylic acid which could be induced by either the pathogen and/or elicitors leading to systemic acquired resistance; the second, mediated by jasmonate and ethylene which was caused by Trichoderma and another beneficial microorganism(s) leading to the induction of systemic acquired resistance. Both types of resistance do not directly affect the pathogen but keep the plant ready for biotic and/or abiotic stresses.

In addition to *Trichoderma* as a biocontrol agent, plant growth-promoting rhizobacteria (PGPR) such as *P. fluorescence* and *Bacillus subtilis* are non-pathogenic rhizobacteria which are also widely used in agricultural production systems to suppress biotic and abiotic stresses [26,27,28]. *P. fluorescence* and *B. subtilis* are the most exploited bacteria for biological control of soil-borne and foliar plant pathogens [29,30]. It has been found that *P. fluorescence* and *B. subtilis* suppress soil-borne pathogens by rhizosphere colonization, antibiosis, iron chelation via siderophore production, and induction of systemic resistance (ISR) [18,31,32].

Studies using *T. viride*, *P. fluorescence*, and *B. subtilis* as biocontrol agents against *R. Solani*-related diseases on coriander plants are rare. Therefore, comparing their efficiencies relative to common conventional fungicides will furnish useful information towards assessing the efficacy of these biocontrol agents to be suitable alternative managing techniques against damping-off and root rot/wilt diseases of coriander. In this study, the effect of three biocontrol agents, *T. viride*, *P. fluorescence*, and *B. subtilis*, on *R. solani* which causes damping-off and root rot/wilt diseases for coriander plants, were evaluated compared to two conventional fungicides. For accomplishing this objective: (i) the antifungal activities of the tested microbes against *R. solani* were determined, (ii) the impact of the tested biocontrol agents versus the conventional fungicides on the growth of coriander seedlings was investigated, and (iii) the physiological up-regulation instigated by the studied biocontrol agents versus conventional fungicides were examined to find out the main defense traits associated with tolerance infection and assess the best bioagent that could be sharing in enhancing the tolerance of coriander plants against *R. solani.*

## 2. Results

### 2.1. Pathogenicity Test

**Figure 1:**
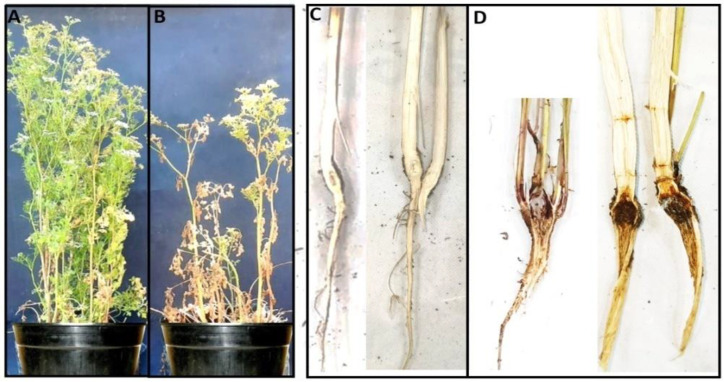
Pathogenicity test of *R. solani* against coriander. (**A**) Healthy uninfected plant, (**B**) root rot wilt symptoms on *R. solani*-infected plants appeared as yellowing, stunting, and wilting. (**C**) Longitudinal section in healthy coriander root, no rot or discoloration were detected. (**D**) Longitudinal section showing root rot/wilt symptoms in infected coriander plants.

*R. solani* denoted pre- and post-emergence damping-off and root rot/wilt symptoms on coriander cv. Baladi compared with the control (Figure 1). *R. solani* was able to infect coriander seeds and impeded the growth of coriander seedlings causing 28.3% pre-emergence damping-off. Additionally, it attacked the emerged seedling and resulted in high, 41.7%, post-emergence damping-off. In addition, *R. solani* infected the growing plants and expressed root rot and wilt symptoms; the incidence of root rot/wilt was estimated as 46.7%, and the severity was approximately 25%.

### 2.2. Half-Maximal Inhibitory Concentration (IC_50_)

**Figure 2:**
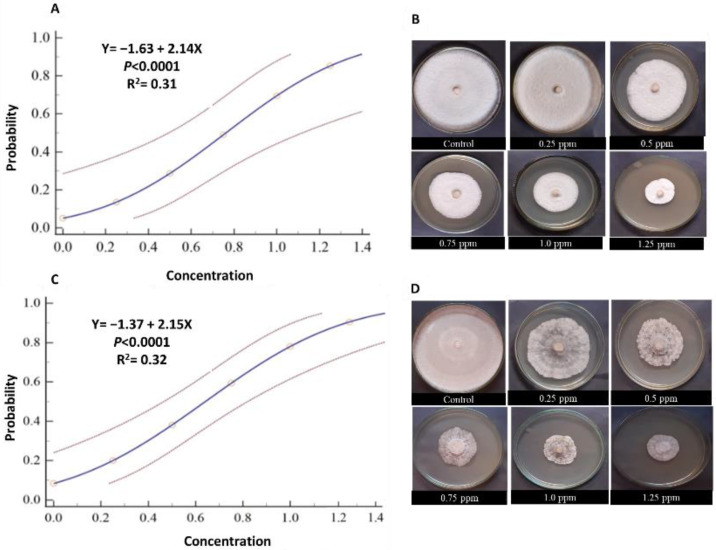
Probit regression analysis of the inhibition effects of Rizolex-T 50% WP (**A**,**B**) and Amistar 25% SC (**C**,**D**) against *R. solani*. Each treatment is represented by five replicates (*n* = 5). The dose–response regression lines are presented as a blue line. R^2^ and *p*-value based on the F test (*p* < 0.05) are shown within the graphs.

The half-maximal inhibitory concentration (IC_50_) of two fungicides, Amistar 25% SC and Rizolex-T 50% WP against *R. solani* was calculated using probit regression analysis (PRA) (Table 1 and Figure 2). The tested fungicides exhibited a high inhibitory effect against *R. solani*. All tested concentrations were able to partially suppress the growth of *R. solani*. The probit analysis showed that the IC_50_ and IC_90_ for Amistar 25% were 0.64 and 1.24 ppm, respectively. Rizolex-TSC showed a similar effect on the growth development of *R. solani*, which expressed IC_50_ and IC_90_ at 0.76 and 1.36 ppm, respectively.

### 2.3. In Vitro Effect of Biocontrol Agents against Pathogenic Fungi

**Figure 3:**
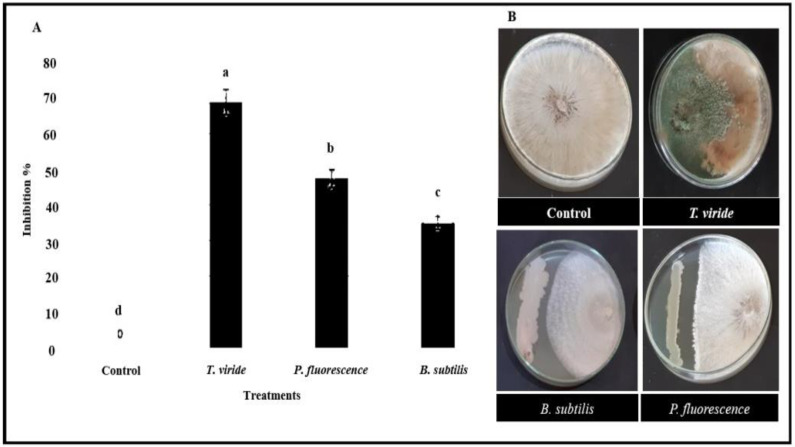
The antagonism between *T. viride*, *P. fluorescence*, and *B. subtilis* and the pathogenic fungi *R. solani* as an inhibitory percentage (**A**), different letters indicate statistically significant differences among treatments, while the same letters signify no significant differences between them according to Tukey’s honestly significant difference test (*p* < 0.05). No inhibition was observed in non-treated *R. solani* compared with noticeable inhibition of the mycelium growth by *T. viride*, *P. fluorescence*, and *B. subtilis* (**B**).

All tested biocontrol agents, *T. viride*, *P. fluorescence*, and *B. subtilis* significantly reduced the linear growth of *R. solani* (Figure 3). *T. viride* showed the highest inhibition percentage, 68.7%, of *R. solani* growth compared to the bacterial bioagents. Comparatively, *P. fluorescence* apparently minimized the mycelium linear growth of *R. solani* to 47.4% compared with the control. On the other hand, *B. subtilis* on mycelium recorded 35% inhibition of the linear growth of *R. solani*.

### 2.4. Antifungal Enzymes (Cell-Wall Degrading Enzymes)

**Figure 4:**
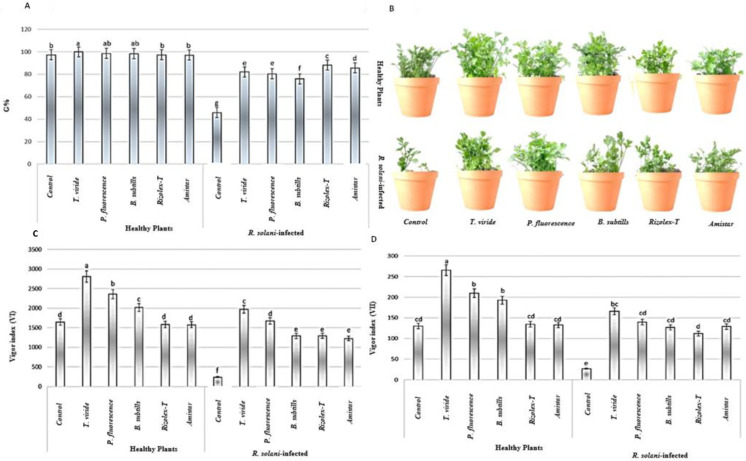
Effects of bioagent treatments on (**A**) the percentage of seed germination, (**B**) damping-off and viability of coriander seedlings, (**C**) the vigor index based on the root and shoot length (VI), (**D**) the vigor index based on seedling fresh and dry weights (VII). Values presented as the means ± the standard deviation of five replicates. The different letters indicate a statistically significant difference between treatments.

The activity of antifungal enzymes was measured in the culture filtrates of *T. viride*, *P. fluorescence*, and *B. subtilis*. The enzyme activities of cellulase, chitinase, and protease are presented in Figure 4. The highest activities of the tested enzymes were observed in culture filtrates of *T. viride*. In this regard, the activities of the cellulose, chitinase, and protease enzyme were 0.064 mg/mL culture filtrate/min, 3.35 μmol of NAG/mL culture filtrate/min, and 0.462 μmol tyrosine/min/mg protein, respectively. However, *P. fluorescence* showed intermediated values of cellulase, chitinase, and protease activities compared to the other two microbes, reaching 0.053 mg/mL culture filtrate/min, 2.72 μmol of NAG/mL/min, and 0.257 μmol tyrosine/min/mg protein, respectively. *B. subtilis* isolate exhibited the lowest enzymes activity, compared with *T. viride* and *P. fluorescence*, which reached 0.032 mg/mL culture filtrate/min, 2.54 μmol of NAG/mL/min, and 0.197 μmol tyrosine/min/mg proteins for cellulase, chitinase, and protease, respectively.

### 2.5. Effect of Bioagents on the Vegetative Traits of the Infected Coriander Plants in Comparison with Traditional Fungicides

**Figure 5:**
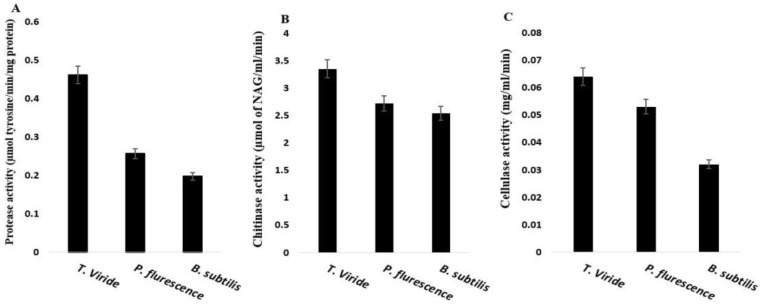
Production of cell-wall degrading enzymes; (**A**) cellulase, (**B**) chitinase, (**C**) protease of *T. viride*, *P. fluorescence*, and *B. subtilis.* Values presented as the means ± the standard deviation of five replicates.

Coriander seedlings infected with *R. solani* showed a significant reduction in germination percentage, seedling length, and weight which led to a higher reduction on the vigor index (VI and VII) Figure 5. The biocontrol agents expressed a significant impact on the germination and the vigor index of non-infected and infected coriander seedlings. All biocontrol agents and tested fungicides significantly increased the G% of infected coriander seeds compared with the infected plants only. However, a noticeable performance of *T. viride* was observed, which showed the highest G% (99.9, 82.2), VI (2807.5, 1972.8), and VII (265.7, 166.2) compared to controls G% (97.3, 45.9), VI (1646.5, 145.2), VII (130, 26.7) for both non-infected and infected plants, respectively. *P. fluorescence* to a large extent had the same effect as *T. viride* on G% and vigor index for infected and non-infected coriander plants, while *B. subtilis* showed the lowest mitigation effect against *R. solani* compared to the other two agents. The data also showed that the conventional fungicides showed a higher G% compared to that of the used biocontrol agents, but the vigor index was lower than that of *T. viride*.

### 2.6. In Vivo Effects of Bioagents on the Causal Pathogen of Coriander Damping-off and Wilt/Root Rot in Comparison with Traditional Fungicides

In general, no disease symptoms were observed in non-infected coriander plants. As presented in Table 2, the application of traditional fungicides and the biocontrol agents significantly alleviated coriander pre- and post-emergence damping-off and root rot/wilt incidence and severity. The obtained data revealed the high efficacy of both fungicides, reducing the pre- and post-emergence damping-off and root rot/wilt severity compared with the three biocontrol agents. There was no significant effect between the two fungicides, except for the pre-emergence damping-off which was 11.8% for Amistar and 14.3% for Rizolex-T. Although all the tested biocontrol agents significantly reduced the coriander root disease caused by *R. solani*, there was a variation between the tested bioagents regarding disease suppression. *T. viride* was more efficient relative to *P. fluorescence* and *B. subtilis* in reducing pre- and post-emergence damping-off and root rot/wilt severity. It reduced the pre-emergence damping-off to 17.8%, followed by *P. fluorescence* at 19.6%, and the lowest for *B. subtilis* 24.1%, compared with 54.1% for the infected control. The tested biocontrol agents significantly minimized the post-emergence damping-off with the same extent recording 11.3%, 12.4%, and 13.5% for *T. viride*, *P. fluorescence*, and *B. subtilis*, respectively, relative to 38.3% of the infected plants. *T. viride* and *P. fluorescence* mostly denoted no significant effect on root rot/wilt severity; both reduced the severity to 7.5 and 8.5%, respectively, compared with 15.1% for the infected plants.

### 2.7. Biochemical Traits of Coriander Plants Treated with Biocontrol Agents versus Fungicides under R. solani Infection

**Figure 6:**
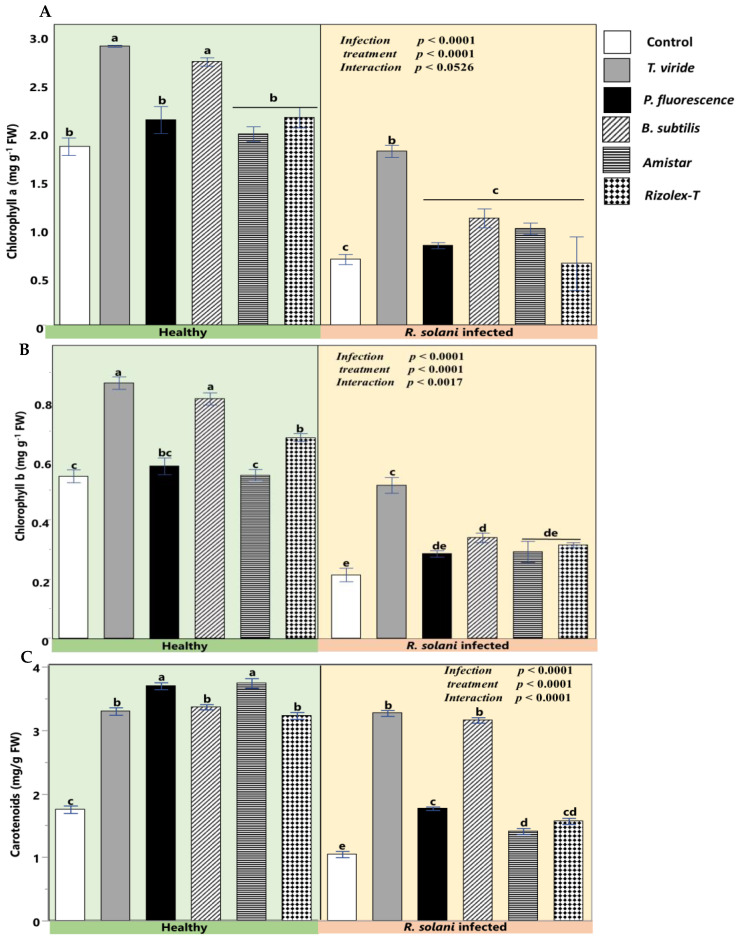
Biocontrol agents’ effect on coriander photosynthetic pigment content under infection by *R. solani.* (**A**) Chlorophyll *a* (mg g^−1^ FW), (**B**) Chlorophyll *b* (mg g^−1^ FW), and (**C**) Carotenoids (mg g^−1^ FW). Data presented are the means ± the standard deviation (mean ± SD) of five biological replicates. Different letters indicate statistically significant differences among treatments, while the same letters signify no significant differences between them according to a Tukey’s honestly significant difference test (*p* < 0.05).

The data of the present work depicted a highly significant decline in Chla, Chlb, and carotenoids by *R. solani*. However, using biocontrol agents (*T. viride*, *P. fluorescence*, and *B. subtilis*) and two fungicides (Rizolex-T50%WP and Amistar 25% SC) exhibited a significant increase in the photosynthetic parameters compared to infected plants. Interestingly, the interactive difference of *R. solani* and biocontrol agents exerted the highest positive role on photosynthesis. Nevertheless, the magnitude of alleviation was higher than the stressed plants only and could not retain the photosynthetic attributes to that of control plants. Compared with conventional fungicides, applying *T. viride* significantly alleviated the photosynthetic pigments in healthy and infected plants. A similar effect was observed with the application of *B. subtilis* on chlorophyll a and b in healthy plants and carotenoids in infected plants. However, no significant effect was observed for *P. fluorescence* compared with fungicides (Figure 6).

**Figure 7:**
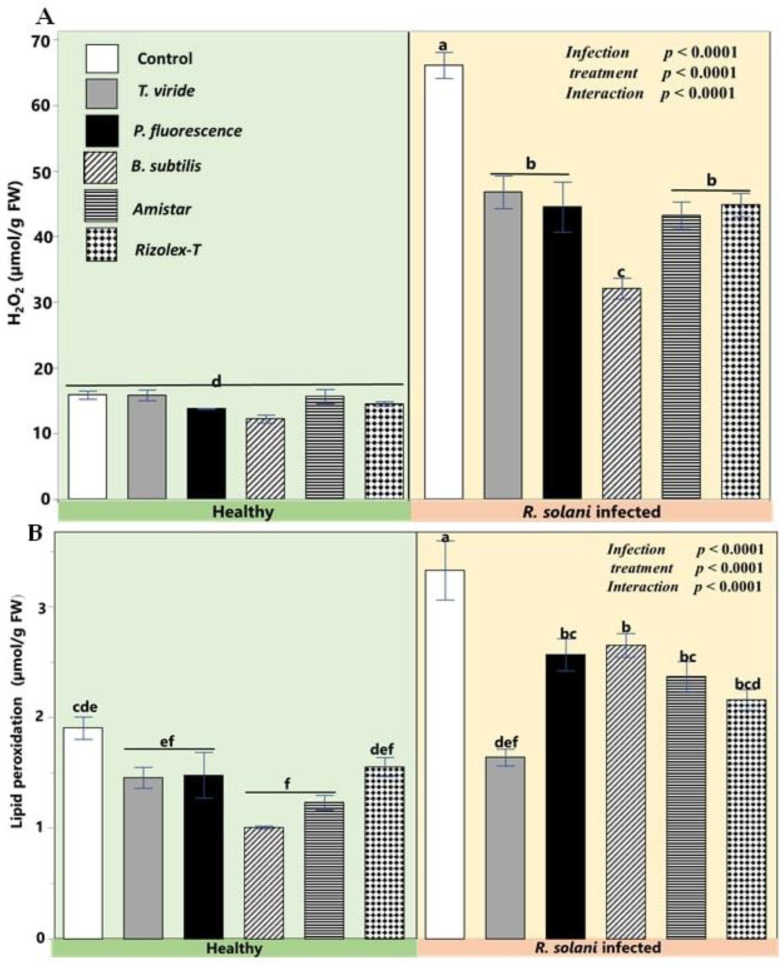
Effects of biocontrol agents on oxidative stress compounds in coriander infected by *R. solani.* (**A**) Hydrogen peroxide (µmol g^−1^ FW), and (**B**) lipid peroxidation (µmol g^−1^ FW). Data presented are the means ± the standard deviation (mean ± SD) of five biological replicates. Different letters indicate statistically significant differences among treatments, while the same letters signify no significant differences between them according to a Tukey’s honestly significant difference test (*p* < 0.05).

As demonstrated in Figure 7, the infection by *R. solani* exerted the most deleterious impacts on coriander plants by increasing the oxidative stress markers such as hydrogen peroxide and lipid peroxidation over the noninfected plants. The different applicants had a non-significant effect on the contents of hydrogen peroxide and lipid peroxidation of the non-stressed plants. However, the effects of various applicants on reducing these oxidative markers became significant when joined with *R. solani* stressed plants. The application of *T. viride* as a biocontrol agent kept the level of lipid peroxidation at the same level of healthy plants while it was significantly high in infected controls. Additionally, infected plants treated with *P. fluorescence* showed significantly lower H2O2 levels than fungicides.

**Figure 8:**
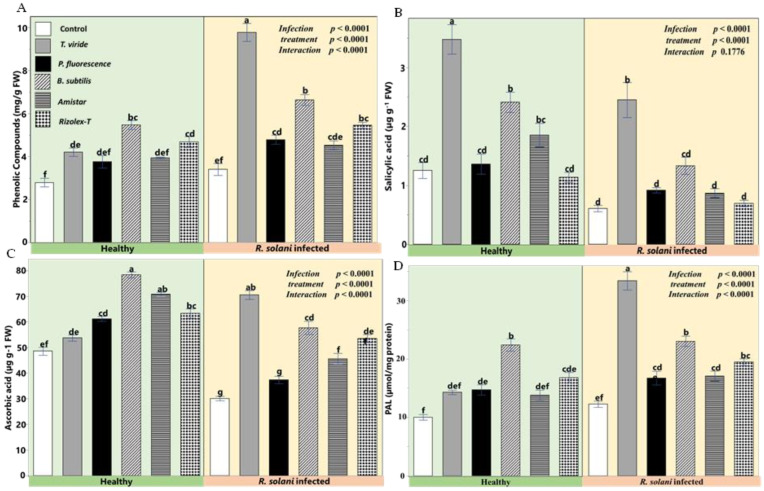
Effects of biocontrol agents on defense-related compounds in coriander infected by *R. solani.* (**A**) Total phenolics (µg g^−1^ FW), (**B**) salicylic acid (µg g^−1^ FW), (**C**) ascorbic acid (ASA) (µg g^−1^ FW), and (**D**) phenylalanine ammonia-lyase (PAL; a key SA biosynthesis enzyme) (µmol mg^−1^ protein). Data presented are the means ± the standard deviation (mean ± SD) of five biological replicates. Different letters indicate statistically significant differences among treatments, while the same letters signify no significant differences between them according toa Tukey’s honestly significant difference test (*p* < 0.05).

The biocontrol agents enhanced the defense-related parameters in terms of phenolics content and the activities of PAL in response to *R. solani*. The application of *T. viride* promoted the activity of defense-related compounds in *R. solani*-infected plants compared to conventional fungicides and the control (Figure 8). Additionally, the salicylic acid (SA) and ascorbic acid (ASA) of the coriander leaves were adversely affected by *R. solani*. At the same time, applying the biocontrol agents improved the content of SA, and a higher improvement was recorded for *T. viride* in healthy and infected plants. Although the application of *B. subtilis* reflected high phenolic compounds, PAL, SA, and ASA compared with *P. fluorescence* and fungicides, the difference was insignificant. Application of *P. fluorescence* significantly increased the phenolic compounds and PAL in infected coriander compared with the control but not compared to the tested fungicides.

**Figure 9:**
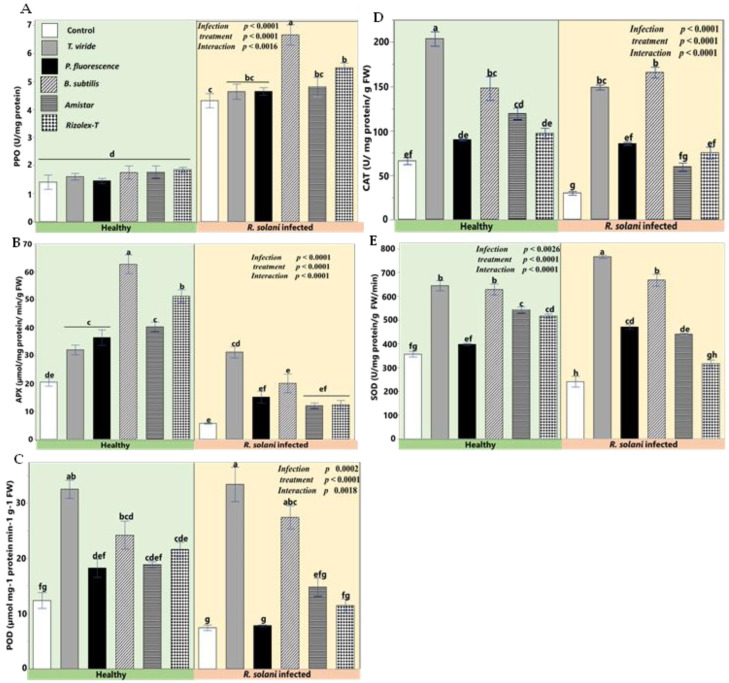
Effects of biocontrol agents on enzymatic antioxidant machinery in infected coriander seedlings. (**A**) Polyphenol oxidase (PPO), (**B**) ascorbate peroxidase (APX), (**C**) guaiacol peroxidase (POD), (**D**) catalase (CAT), (**E**) superoxide dismutase (SOD). Data presented are the means ± the standard deviation (mean ± SD) of five biological replicates. Different letters indicate statistically significant differences among treatments, while the same letters signify no significant differences between them according to a Tukey’s honestly significant difference test (*p* < 0.05).

In response to *R. solani*-infected plants, the contents or activities of the antioxidant system, i.e., APX, CAT, SOD, and POD, were significantly reduced compared to non-stressed plants; especially that of APX and CAT (Figure 9). Various applicants under natural conditions had highly significant increments in their values. The potential stimulatory effect of biocontrol agents and fungicides under infection conditions on antioxidants was evident to multiple degrees. In this sense, the biocontrol agents’ treatments to stressed plants augmented the antioxidants and increased their values higher than the control plants. Applying *T. viride* and *B. subtilis* in *R. solani*-infected plants increased the activities of the antioxidant system higher than fungicides, except for PPO *T. viride* which showed a similar effect to fungicides. Infected plants treated with *P. fluorescence* showed high CAT, APX, SOD, and POD compared with the control, but it did not show significant differences compared to conventional fungicides.

### 2.8. Heatmap and Principal Component Analysis (PCA) Correlation of the Physiological Data under Different Treatments

**Figure 10:**
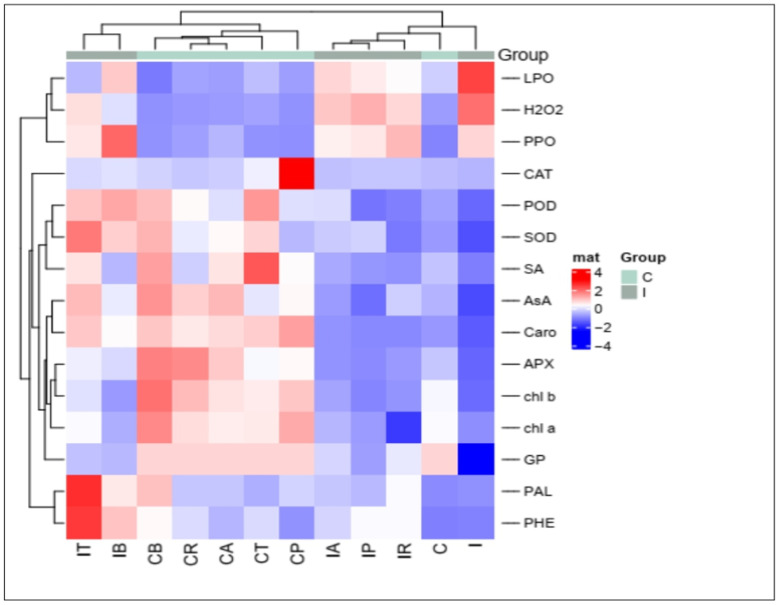
Heatmap analyses for physiological traits of healthy and *R. solani*-infected coriander treated with biocontrol agents.

**Figure 11:**
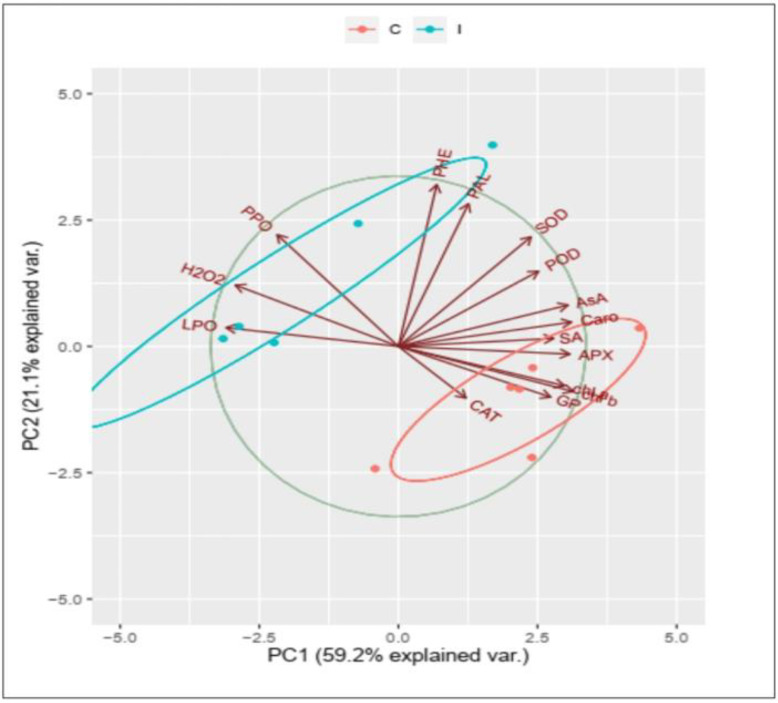
Principal component analysis (PCA) correlation of physiological traits of healthy and *R. solani*-infected coriander treated with biocontrol agents.

All mean values of the physiological traits under various treatments were subjected to hierarchical clustering as a heatmap (Figure 10) and correlation analysis as PCA (Figure 11). The heat map analysis of data correlation revealed the role of the used biocontrol agents in the upregulation of the physiological responses of the studied plants, especially that of the infected plants using the biocontrol agents; especially *T. viride* compared to the infected plants only. Thus, we found five groups that have the same column correlation and are arranged from right to left as follows:(i) infected plants, (ii) control plants, (iii) the infected plants + *P. fluorescence*, infected plants + Rhizolex, and infected plants + Amistar, (iv) included five treatments, i.e., the control plants treated with fungicides and biocontrol agents, (v) the highest upregulation was denoted for the fifth group of infected plants + *B. subtilis*, and infected plants + *T. viride*. The data showed that downregulation of pigments (Chl*a*, Chl*b*, and carotenoids), antioxidants (ASA, CAT, APX, and SOD), and defense-related traits (SA, Phen, and PAL) have a negative correlation under *R. solani*. At the same time, the data of oxidative damage (H_2_O_2_ and lipid peroxidation) and PPO activity positively correlate with the infection.

On the other hand, the most striking tolerance exerted by the used biocontrol agents, especially *T. viride* and *B. subtilis*, reacted positively by enhancing the level of phenolic compounds and PAL, antioxidants, and SA and negatively by reducing lipid peroxidation, H_2_O_2_, and PPO. The PCA of the studied physiological traits showed that two components, PC1 (contributed 59.2% from the whole dataset) and PC2 (sharing 21.1% from the entire dataset), collectively ascribed 90.3% for data variability. Furthermore, it was found that the variables of H_2_O_2_, lipid peroxidation, and PPO were strongly connected with stressed plants without any recovery agents. While the variables (CAT, APX, SOD, SA, and Chl*a*) were strongly associated with the upregulation mechanism under various protecting agents.

## 3. Discussion

The study investigated the seed application of three biocontrol agents, *T. viride*, *P. fluorescence*, and *B. subtilis* against *R. solani*, the causal agent of coriander damping-off and root rot/wilt diseases. The bio-agents’ efficacy was tested in vitro and in vivo compared with the two conventional fungicides Amistar 25% SC and Rizolex-T 50% WP at 90% inhibitory concentration (IC_90_). The results revealed that the tested biocontrol agents exhibited significant inhibition of *R. solani* growth. *T. viride* resulted in the highest inhibition percentages, followed by *P. fluorescence* and then *B. subtilis*. The inhibitory effect of the tested biocontrol agents may be due to the secretion of direct and indirect antifungal enzymes and compounds. Our results detected a high level of cellulase, chitinase, and protease activities in *T. viride*, *P. fluorescence*, and *B. subtilis* culture filtrates. Kubicek et al. [33] stated that hydrolytic enzymes, such as chitinases, cellulase, and β-1,3-glucanases, play a crucial role in degrading pathogenic fungal cell walls and inhibiting fungal growth. These antifungal compounds target the fungal cell wall, which is composed mainly, 90%, of the polysaccharides chitin, β-(1,3)-, β-(1,4)- and β-(1,6)-glucans, chitosan, mannan, α-glucans, and galactomannan, as well as proteins [34]. In addition, the fungal proteases contribute to fungal-cell-wall lysis [35]. The ability of *Trichoderma* spp. to secret extracellular enzymes is known. It produces extracellular enzymes that hydrolyze the basic components of the pathogenic fungi cell wall, such as chitinases which hydrolyze the β-glycosidic in chitin, proteases, and β-(1,3)- and β-(1,6)-glucanases [36,37].

The antifungal compounds in the culture filtrate of *B. subtilis* increased the cytoplasmic vacuoles, cell-wall disintegration, and protoplasm leaks in the mycelium of *R. solani* [38]. In addition, it was reported in previous studies that different fungal cell-wall-degrading enzymes, i.e., chitinase and *β*-1,3-glucanase, may be responsible for the antagonistic activity of *P. fluorescens* against *R. solani* [39,40,41]. *P. fluorescens* was also reported to produce 2,4-diacetyl phloroglucinol which also contributes to the suppression of *R. solani* [42] and can produce antibiotics and HCN, which suppresses wheat infection by *Septoria tritici* and *Puccinia recondita* f. sp. *tritici* [43]. On the other hand, Bacillus can produce a variety of microbial plant bio stimulants to trigger or increase plant defense response, promote plant growth, and improve their response to different stresses. The induction of systemic resistance (ISR) is a primary factor involved in the suppression of plant pathogens by plant growth-promoting rhizobacteria [44] as has been reported for *Bacillus amyloliquefaciens* FZB42 and *Bacillus cereus* AR156 [45]. Hence, the reduction of pre- and post-emergence and wilt disease of *R. solani* by the applied biocontrol agents may be associated with the aforementioned mechanisms. Interestingly, the different activities of chitinases, cellulase, and β-1,3-glucanases were associated with their various impacts on *R. solani* where the highest antimicrobial agents for *T. viride* correlated to its high inhibition of *R. solani* proliferation relative to *P. fluorescens* which was intermediate, and *B. cereus* which had the lowest microbe impact on the pathogen infection.

In the present investigation, the efficacy of the tested biocontrol agents against *R. solani* was highly observed in the pot experiment. All biocontrol agents significantly alleviated damping-off and root rot/wilt incidence and severity caused by *R. solani* compared with the control. Although Amistar 25% SC was the most effective in enhancing G% compared with biocontrol agents, the biocontrol agents showed significantly higher vigor index (VI) values on healthy and infected coriander seedlings; especially that of *T. viride*. It has been stated that *Trichoderma* spp. secrete various secondary metabolites and growth-promoting substances for plants that have the same effect as auxin, thereby enhancing plant development [23,46,47]. By measuring the physiological traits of infected coriander plants, the damaging impacts of *R. solani* were associated with the reduction of photosynthetic performance in terms of Chl*a*, Chl*b*, and carotenoids. Using the biocontrol agents improved seedling viability by increasing the contents of photosynthetic pigments compared with control and fungicides treatment. These data went in parallel with Cai et al. [48], who reported that the promotion of rice growth and the enhancement of plant viability due to *Trichoderma* treatment could be linked to increasing the efficacy of photosynthesis. Furthermore, in the present study *P. fluorescence* was the second-most effective treatment for increasing plant viability and vigor index in healthy and infected coriander plants. Improvement of coriander growth and vigor index resulting from *P. fluorescens* might be due to additional features that are not tested in this study such as the enhancement of mineral uptake, production of siderophores (i.e., pyoverdine), plant growth hormones such as ethylene, and other enzymes [49,50]. The data of PCA and heatmap analyses suggested the positive correlation of pigments with the healthiness of plants under infection, especially Chl*a* and Chl*b*, and the high ability of *T. viride* to boost pigment content under natural or infection conditions.

A high production of reactive oxygen species (ROS; i.e., H_2_O_2_) was observed in coriander treated with *R. solani*. Similarly, Youssef et al. [51] reported that the damaging impact of *R. solani* on the infected coriander seedlings could be due to the spreading of disease and generation of ROS. The spreading of the disease may be associated with the role of ROS in damaging the plant membrane which lessens membrane stability and facilitates the pathogen’s invasion. In the same way, the coriander seedlings suffered from severe membrane damage in terms of lipid peroxidation, allowing easy pathogen transfer in host tissues. On the other hand, the applied biocontrol agents were able to protect the coriander cells and suppress excessive production of lipid peroxidation parallel to the reduction of H_2_O_2_ similar to that reported by Bagy et al. [52]. Several studies recommended that the stabilization of cell membranes is known to be correlated with abiotic and biotic stress tolerance [53,54,55,56,57]. Thus, membrane stabilization and reactive oxygen species are essential traits associated with pathogen severity and disease incidence. PCA and heatmap analyses suggested the positive association of infection severity with increasing levels of H_2_O_2_ and lipid peroxidation. Additionally, heatmap analysis indicated the positive correlation of controlling disease severity by the protecting agents, especially *T. viride,* with the attenuation of H_2_O_2_ and lipid peroxidation in the infected plants.

Biocontrol agents mediated mechanisms that restrict ROS and oxidative stress bursts at the cellular level. The restriction of ROS experienced in infected plants treated with *T. viride*, *P. fluorescence*, and *B. subtilis* could be ascribed to a high accumulation of antioxidative molecules and activities. The PCA and heatmap analyses suggested that CAT, APX, SOD, and POD are associated with the tolerance of plants against infection, and a clear positive correlation was also recorded when the non-infected plants were treated with various agents, especially *Trichoderma*. Studies by Chowdappa et al. [58] and Kumar et al. [59] showed that oxidative stress was regulated in infected plants through the increase in antioxidant enzymes such as POD, CAT, and SOD under different *Trichoderma* species. Similar results have also been reported in *T. harzianum* by Youssef et al. [51] and in *T. atroviride* by Nawrocka et al. [60]. Moreover, Chandrasekaran and Chun, [61] stated that *B. subtilis* increased the activity of antioxidant enzymes (SOD, POD, and CAT), which subsequently alleviated the ISR oxidative stress in tomato leaves and expressed resistance against bacterial soft rot. The plants are rich in aromatic secondary compounds such asphenolic compounds, quinones, flavonoids, tannins, and coumarins. These metabolites exhibited antimicrobial activities acting as plant defense mechanisms against pathogen infection [52,62]. In the present study, the phenylpropanoid pathway was significantly up-regulated. The secondary metabolites relevant to plant survival were elucidated in *Arabidopsis* and other species [62,63,64].

Compared with the control, a high accumulation of resistance-related compounds and enzymes, e.g., phenolic compounds, phenylalanine ammonia-lyase, and polyphenol oxidase, was observed when *R. solani*-infected coriander was treated with biocontrol agents. In the PCA analysis, *R. solani* showed a negative correlation with the phenolic content of coriander plants. Reducing phenolics was one of the significant causes of disease severity induced by *R. solani*. This tendency is in line with the data of PAL activity as phenolic-biosynthesizing enzymes were lessened by pathogen infection and up-regulated with bioagent application. Interestingly, a positive effect was noticed in the heatmap correlation diagram between the increment of phenolic compounds and PAL activity and the interactive development of biocontrol agents and the infected coriander plants, which was more pronounced for *Trichoderma*-treated plants. In this sense, several studies reported that the protective action of *Trichoderma* may go back to the ability to produce many secondary metabolites [65,66,67,68]. These results match a study by Jaroszuk-Ściseł et al. [69], who found a significant increase in the activity of the phenylalanine and tyrosine lyase (TAL), catalase, guaiacol peroxidase, as well as glucanase and chitinase (PR proteins) in wheat plants when treated with *Trichoderma*. It was reported by Jain et al. [70] that high production of defense-related enzymes such as PPO, POD, and PAL was expressed in soybean root tissue after treatment with *Bacillus*. Additionally, a study by Chandrasekaran and Chun [61] stated that *B. subtilis* increased the activity of PPO and PAL, which subsequently alleviated the induced systemic resistance to oxidative stress in tomato leaves and expressed resistance against bacterial soft rot. Therefore, the reduction in pathogen proliferation by the use of microbial biocontrol agents may be attributed to their antimicrobial properties and instigation of host-defense mechanism which suppressed the growth incidence of *R. solani* on coriander plants.

SA is a crucial signaling molecule that induces plant defense mechanisms against various pathogens and induction of systematic resistance. It confers a long-lasting, broad-spectrum resistance against pathogen infection [63]. Generally, *T. viride*, *P. fluorescence*, and *B. subtilis* induced the resistance of coriander via SA signaling pathways. In this respect, coriander plants treated with these agents had increased levels of SA under *R. solani,* which is associated with a reduction in disease severity and infection incidence. This conclusion was concomitant with a positive correlation of SA with plant resistance against infection. On the other hand, *R. solani*-infected plants reduced SA production, causing an increment in disease severity. The protective action of *Trichoderma* may go back to its role in mediating the signal pathways of ethylene/jasmonate and salicylic acid [71]. Niu et al. [72] stated that *B. cereus*-induced *Arabidopsis* systematically acquired resistance through the SA signaling pathway to enhance its disease resistance. Plant growth-promoting microbes activate the SA-signaling pathway that depends on the up-regulation of the PR-1 gene [73]. Against *Botrytis cinerea*, various *Bacillus* species could pre-activate systemic resistance through different mechanisms [63,74]. Additionally, *P. fluorescens* induces plant resistance against pathogenic microbes by producing SA in the rhizosphere [75,76].

## 4. Material and Methods

### 4.1. Isolate Source, Identification, and Pathogenicity

The *R. solani* isolate (Accession No. OP108814) used in this study was selected because of its high frequency and virulence to coriander compared to the other pathogenic fungal collection based on Lessy et al. [77]. The isolate showed 100% pairwise identity with KM013470 which belongs to the anastomosis group (AG-4) [78]. It was isolated from wilted coriander plants collected from different fields in Minia province, Egypt. The isolate was identified by the Molecular Biology Research Unit, Assiut University and SolGent Company, Daejeon South Korea based on sequence analysis of the internal transcribed spacer (ITS) region using ITS1 and ITS4 primers. PCR was performed using ITS1 (forward) and ITS4 (reverse) primers incorporated in the reaction mixture. Primers have the following compositions: ITS1 (5′-TCC GTA GGT GAA CCT GCG G-3′), and ITS4 (5′-TCC TCC GCT TAT TGA TAT GC-3′). The isolate was kept on potato dextrose agar (PDA) (Difco, Sparks, MD, USA) at 25 ± 2 °C during the study and at −20 °C for long-term storage.

The pathogenicity of *R. solani* isolate was carried out under the greenhouse conditions of the Experimental Farm of the Plant Pathology Department, Faculty of Agriculture, Minia University, using coriander seeds (cv. Balady) obtained from the Agricultural Research Center, Giza, Egypt.

Inoculum of *R. solani* isolate was prepared, according to Al-Fadhal et al. [79] with minor modifications, using a five-millimeter diameter disk of a five-day-old culture of the fungal isolate grown on sterilized barley grains. Inoculated flasks were kept at 25 ± 2 °C for two weeks and then were used for soil infestation.

Soil infestation was done seven days before planting by thoroughly mixing 2% of the inoculum with soil (W:W), representing a barley culture of a single fungus with the soil in the pot. The infested soil was irrigated daily till planting. Surface-disinfected seeds of coriander were used for sowing. Five replicates (pots) were sown with five coriander seeds producing ten seedlings. Sterilized and non-inoculated barley medium was used in the check control treatment. The pots were watered when necessary. Plants were regularly examined for disease symptoms. Pre- and post-emergence damping-off symptoms were calculated after 2 and 4 weeks, respectively, as follows:

$$\mathrm{Disease}\;\mathrm{incidence}=\frac{\mathrm{No}.\;\mathrm{of}\;\mathrm{infected}\;\mathrm{seedlinges}}{\mathrm{Total}\;\mathrm{no}.\;\mathrm{ofseedlings}}\times 100$$

The severity of wilt/root rot diseases was rated after 6 weeks using the indexing method as described by Beale et al. [80]. The roots of coriander plants were washed and divided into five categories according to the percentage of all roots with lesions typical of root rot: zero (0) trace to 10% (1), >10% and ≤30% (2), >30% and ≤60% (3), and >60% (4).

$$\mathrm{Disease}\;\mathrm{severity}\;\left(\%\right)=\frac{\sum \;\left(\mathrm{rating}\;\mathrm{no}.\times \;\mathrm{no}.\;\mathrm{of}\;\mathrm{plants}\;\mathrm{in}\;\mathrm{the}\;\mathrm{rating}\right)}{\left(\mathrm{total}\;\mathrm{no}.\;\mathrm{of}\;\mathrm{plants}\;\times \;\mathrm{highest}\;\mathrm{rating}\right)}\times 100$$

### 4.2. Half-Maximal Inhibitory Concentration (IC_50_)

The half-maximal inhibitory concentration (IC_50_) of two fungicides, Rizolex-T 50% wettable powder (WP) [20% 0-(2,6–Dichloro-4-methylphenyl)-0,0-dimethyl phosphorothioateand 30% Tetramethyl thiuram disulfide; bis (dimethyl thiocarbamoyl) disulfide] and Amistar 25% suspension concentrate (SC) (Azoxystrobin 25%) against *R. solani* was calculated using probit regression analysis (PRA). The probit regression analysis (PRA) was used to fit the probit/logit sigmoid dose–response curves and to calculate inhibitory concentrations (IC_50_ and IC_90_) with 95% confidence intervals [81]. The serial concentration of each fungicide 0.25, 0.5, 0.75, 1.0, and 1.25 ppm was tested by amending each fungicide into the PDA media with the concentration adjusted as mentioned above. The media was poured into 9 cm Petri plates, and a 3 mm disk of *R. solani* culture was transferred to the center of the Petri plates. Only PDA was used for controls. The plates were incubated for five days at 25 °C. The linear growth of each isolate was measured. The inhibition percentage was calculated as follows:

Percent inhibition = [(Linear growth of control − Linear growth of treated)/linear growth of control] × 100.

### 4.3. In Vitro Effect of Trichoderma Viride against R. solani

*T. viride* isolate No. FUE19 was provided by Dr. Nada Hemada, Genetics Department, Faculty of Agriculture, Fayoum University. The isolate was recovered from the rhizosphere soil of cucumber [82]. *T. viride* was maintained on PDA media and incubated for six days at 20 °C. The antagonistic effects of *T. viride* were performed according to the method adopted by Perveen and Bokhari [83]. A 5-mm diameter disc from the five-day-old *T. viride* culture was inoculated on PDA on one side of the Petri plates (2 cm away from the edge) and a 5-mm disc obtained from a seven-day-old PDA culture of fungal pathogenic isolate *R. solani*. The disk of each pathogenic isolate was placed on the opposite side of the plate perpendicular to the biocontrol agents and incubated at 25 ± 2 °C for five days. Petri dishes inoculated with pure single pathogenic fungal discs (5 mm diameter) served as the control. Five replications were used for each fungus. Observations on the width of the inhibition zone and mycelia growth of the tested pathogens were recorded, and the percentage of the pathogen growth inhibition was calculated by using the formula proposed by Singh et al. [84].

Growth inhibition percent (GI, %) = C − T/C ×100.

C = growth diameter of the control plate and T = growth diameter of the treated plate.

### 4.4. In Vitro Effect of Pseudomonas fluorescence and Bacillus subtilis against R. solani

The two isolates of *P. fluorescence* and *Bacillus subtilis* were provided by Dr. Marzouk Abdellatif, Department of Plant Pathology at Minia University, Egypt. The bacterial isolates were maintained on PDA for 48 h at 37 °C. The antagonistic effects of the used biocontrol agents were performed according to the methods adopted by Perveen and Bokhari [83]. Loop from the growth of bacteria, two days old, was inoculated on PDA on one side of the Petri plates (2 cm away from the edge), and 5-mm discs obtained from seven-day-old PDA cultures of *R. solani* were placed at the opposite side of the plates perpendicular to the biocontrol agents and incubated at 25 ± 2 °C for five days. Petri dishes inoculated with pure single pathogenic fungal discs (5 mm diameter) served as the control. Five replicates were used for each treatment. Observations on the width of the inhibition zone and mycelia growth of the tested pathogen were recorded. The percentage of pathogen growth inhibition was calculated using the formula proposed by Singh et al. [84], as mentioned previously.

### 4.5. Detecting the Antifungal Enzyme Activity in Biocontrol Agent Culture Filtrates

For enzyme assay, culture filtrates of *Trichoderma viride* were prepared by placing a 3 mm disk of five-day-old culture into 200 mL of nutrient broth medium (NB) and incubating at 25 ± 1 °C for two weeks. The obtained liquid culture was filtered using a syringe filter of 0.22 μm. The culture filtrates of *P. fluorescence* and *B. subtilis* were prepared as described by Sukalpa et al. [85] with minor modifications. *P. fluorescence* and *B. subtilis* were streaked on the solidified King’s B medium and nutrient agar (NA) medium, respectively. The media were then incubated at 28 °C for 24 h [86]. A loop full of bacteria was transferred into a 250 mL conical flask containing a nutrient broth medium. The inoculated flasks were incubated for 48 h on an electric shaker at 200 rpm at 35 ± 1 °C [87]. The obtained culture was centrifuged at 6000 rpm for 10 min and filtered using 0.20 and 0.45 membrane filters.

#### 4.5.1. Chitinase Activity Assay

Chitinase activity was measured for the three biological agents. The extraction was prepared as described previously. Colloidal chitin was selected as the substrate. The reaction of all tested biocontrol agents was prepared as 0.5 mL of 1% *w*/*v* colloidal chitin and 0.5 mL of the enzyme as a control and the tested biocontrol agent extract. The solution was incubated for 12 h at 37 °C. Then, 3 mL of 3, 5-dinitrosalicylic acid reagent was added to stop the reaction, followed by heating at 100 °C for 5 min. The solution was centrifuged, and the reducing sugar in the supernatant was determined as described by Miller [88] where the absorbance was measured at 530 nm using a UV spectrophotometer along with substrate and blanks. One unit (U) of the chitinase activity was used as the amount of enzyme required for the formation of 1 μ mole of the N-acetyl-glucosamine in 1 mL of the reaction under the standard assay conditions [89].

#### 4.5.2. Activity of Cellulase

The cellulase activity was assayed by incubating 1 mL of culture filtrate of each biocontrol agent with 1 mL of 0.5 mM sodium citrate buffer, pH 4.8, and 1 mL of 1% (*w*/*v*) carboxymethyl cellulose at 50 °C for 10 min [90]. The reaction was stopped by the addition of alkaline dinitro salicylic acid [88], and absorbance was read at 540 nm.

#### 4.5.3. Assay of Protease

A reaction mixture containing 0.8 mL of Hammerstein casein (6.0 gL^−1^, dissolved in 0.05 M phosphate buffer, pH 6.0) and 0.2 mL of culture filtrate of each biocontrol agent was incubated without shaking at 37 °C for 15 min. The sample was then centrifuged at 1000× *g* for 10 min. An amount of 1 mL of supernatant was used for the assay of tyrosine, according to Lowry et al. [91]. One unit of enzyme activity was defined as the amount of enzyme required for the format of μmol of tyrosine/min/mg protein.

### 4.6. In Vivo Effect of Bio-Control Agents on the Causal Pathogens of Coriander Damping-Off and Wilt/Root Rot in Comparison with Traditional Fungicides

The experiment was conducted to evaluate the damping-off and root rot/wilt of coriander artificially infected with *R. solani* in response to three biocontrol agents (*T. viride*, *P. fluorescence*, and *B. subtilis*) and two conventional fungicides (Rizolex-T 50% WP and Amistar 25% SC). The experiment was conducted in two trials, and only one trial was presented in this study as there were no significant differences between the trials. Pot experiments were carried out in the greenhouse of the Plant Pathology Department, Faculty of Agriculture, Minia University, during the winter seasons of 2020/2021. The experiment was designed as randomized complete blocks. The preparations of *R. solani* inoculum and soil inoculation were carried out as described in the pathogenicity test. Soil infestation was carried out seven days before planting, and the pots were irrigated every two days until sowing. Sterilized and non-inoculated barley medium was used in the check control treatment.

The experiment was composed of six treatments: control, the three biocontrol agents, and two treatments of conventional fungicides under *R. solani*-infected soils and non-treated soils, thus 12 groups were produced. Surface sterilized coriander seeds were soaked for 2 h before sowing in a suspension of each biocontrol agent separately: *T. harzianum* (5 × 10^6^ CFU/mL) [92], *B. subtilis* (1 × 10^8^ bacterial CFU/mL), and *P. fluorescens* suspension (9 × 10^8^ CFU/mL) (*v*/*w*) [93,94]. For fungicide treatments, coriander seeds were soaked in the IC_90_ of each fungicide (Rizolex-T 50% WP and Amistar 25% SC). Each treatment was represented by 5 replicates: each consisted of 5 pots, and each pot contained 10 seeds which produced 20 seedlings. The experiment was composed of 6 groups of seeds sown in infected soils with *R. solani*. Another 6 groups of seeds were sown in soils that did not receive *R. solani*. Seeds soaked in a particular solution received 30 mL/pot of that solution after two weeks of sowing as post-emergence supplementary application. The control pots received sterilized water at 30 mL/pot.

Four weeks after sowing, the percentage of germinated seeds, roots and shoots length, and vigor index of coriander seedlings were measured using the following equations:

Germination Percentage (GP%) = (Number of germinated seeds/Number of total seeds) × 100

The length of shoots and roots (cm) for coriander plants was used for calculating the vigor index. Ten plants were taken from each replicate. The vigor index based on the root and shoot length (VIL) was measured by the formula described by Abdul-Baki and Anderson [95] as follows:

VIL = (shoot length + root length) × germination (%)

Likewise, the coriander vigor index based on the weight was calculated using the following equation:

VIW = seedling dry mass at the end of the test × germination (%)

Additionally, the pre- and post-emergence damping-off and wilt/root rot disease were estimated after 2, 4, and 6 weeks as described previously.

### 4.7. Physiological Trait Assessment

From the previously mentioned twelve groups, leaves of six-week-old coriander seedlings (the second leaf from the top) were used for the physiological measurements with five biological replicates for each trait. Fresh leaves were immersed in liquid nitrogen and then kept in a deep freeze (−70) until use. Chemicals used for the physiological assessment were previously ordered from LANXESS AG (Kennedyplatz, Essen, Germany).

#### 4.7.1. Pigment Content

Chlorophyll a, b, and carotenoids were recorded from fresh leaves (0.05 g) suspended with 5 mL ethyl alcohol (95%) using equations recommended by Lichtenthaler [96]. The suspended leaves were heated at 60–70 °C until the leaves became colorless. The absorbance readings of the green color produced were monitored with a spectrophotometer (Unico UV-2100 spectrophotometer) at 663, 644, and 452 nm to detect Chl a, Chl b, and carotenoids, respectively.

#### 4.7.2. Oxidative Damage Traits

The foliar content of H_2_O_2_ in coriander leaves was quantified spectrophotometrically using the method described by Mukherjee and Choudhuri [97]. The supernatant of homogenized fresh coriander leaves (0.05 g) in cold acetone was mixed with titanium dioxide-H_2_SO_4_ reagent (20%), and the mixture was centrifuged at 6000 rpm (K centrifuge, Harmonic Series) for 15 min. The intensity of the yellow color of the supernatant was measured at 415 nm using the UV–VIS Spectrophotometer UNICO (Leicestershire, UK). Lipid peroxidation was detected in shoots using the thiobarbituric acid reaction by monitoring malondialdehyde formation as explained by Madhava Rao and Sresty [98] with some modifications.

#### 4.7.3. Resistance-Related Compounds/Activities

##### Estimation of Salicylic Acid (SA)

The concentration of SA in coriander leaves was measured according to the method described by Warrier et al. [99]. Coriander leaves (50 mg) were ground into powder using liquid nitrogen. The powder was homogenized in 80% ethyl alcohol and then centrifuged. The supernatant was cooled on ice for SA measurement. A total of 0.1 mL of the supernatant was mixed with 0.1% ferric chloride (freshly prepared). The violet color of the complex formed between Fe^3+^ ion and SA was detected at 540 nm.

##### Estimation of Phenolic Compounds (Phen)

The concentration of phenolic compounds in coriander leaves was determined based on the method of Kofalvi and Nassuth [100] using a standard curve of gallic acid expressed as mg/g FW. Fresh coriander leaves (0.3 g) were added to methanol (50%) and incubated in a water bath for 1 h at 70 °C. The methanolic extract was mixed with distilled water + Folin Ciocalteu’s reagent + Na_2_CO_3_ at room temperature. After 20 min, absorbance at 725 nm was measured.

##### Phenylalanine Ammonia-Lyase (PAL/EC 4.3.1.5) Activity Assay

PAL activity was examined using the protocol of Havir and Hanson [101]. The supernatant of frozen coriander leaves (0.5 g) homogenized in extraction buffer (5 mL) containing potassium phosphate buffer (100 mM, pH 7.5), EDTA, and PVP was used as an enzyme extract. Aliquots of the extract mixed with borate buffer (80 mM, pH 8.9) and phenylalanine were incubated for one h at 30 °C and then 2 M HCl was added before the concentration of trans-cinnamic acid was measured at 290 nm. The enzyme activity was expressed as μmol/mg protein.

##### Polyphenol Oxidase (PPO/EC 1.10.3.1) Activity Assay

PPO activity was determined using the protocol of Kumar and Khan [102]. The assay mixture for PPO containing phosphate buffer (100 mM, pH 6), catechol, and enzyme extract was incubated for 5 min at 25 °C before the reaction was stopped by adding H_2_SO_4_. Purpurogallin production was measured at 495 nm, and the enzyme activity was expressed as U mg^−1^ protein.

#### 4.7.4. Antioxidant-Response Molecules/Activities

##### Estimation of Superoxide Dismutase (SOD/EC.1.15.1.1) Activity

SOD activity was quantified following the autoxidation of epinephrine, as mentioned by Misra and Fridovich [103], in a reaction medium containing sodium carbonate buffer, EDTA, enzyme extract, and epinephrine. The change in absorbance was monitored at 480 nm for 1 min.

##### Estimation of Catalase (CAT/EC.1.11.1.6) Activity

CAT activity was calculated following the breakdown of H_2_O_2_ for 1 min, where the decrease in absorbance was monitored at 240 nm using the protocol of Noctor et al. [104].

##### Estimation of Ascorbate Peroxidase (APX/EC.1.11.1.11) Activity

The APX activity was detected in previously prepared enzyme extract by monitoring the oxidation of ascorbate as a substrate in the presence of EDTA and H_2_O_2_ at 290 nm using the protocol of Silva et al. [105].

##### Estimation of Guaiacol Peroxidase (POD/EC 1.11.1.7) Activity

The POD activity was determined based on the protocol of Tatiana et al. [106]. The production of tetra guaiacol was monitored in a reaction mixture containing 100 μL enzyme extract, 30 mM potassium phosphate (pH 7), 6.5 mM H_2_O_2,_ and 1.5 mM guaiacol for 1 min at 470 nm.

### 4.8. Statistical Analyses

All lab experiments were conducted using a complete randomized design (CRD). All greenhouse experiments were designed as completely randomized blocks using *R. solani*-infection (healthy vs. infected) as main plots and biocontrol agents and fungicide treatments as subplots. All experiments were repeated twice, with five replicates for each treatment. As the values of each pair of repeated experiments were highly similar, only one experiment’s data were presented. The analysis of variance (ANOVA) was used to test the significant differences among different infection and treatment levels as well as the interaction. A Tukey’s honestly significant difference (HSD) test was used for posthoc analysis. The least significant difference was used to compare the means at *p* ≤ 0.05. The data were analyzed using JMP data analysis software version 14. The principal component analysis (PCA) and heatmap analyses were performed using software applied on http://www.bioinformatics.com.cn/cgi-bin/guide.cgi. The data was accessed on 22 December 2022.

## 5. Conclusions

In conclusion, the in vitro application of *T. viride*, *P. fluorescence*, and *B. subtilis* against *R. solani* significantly inhibited the mycelium growth of *R. solani*. In addition, the activity of antimicrobial enzymes (cellulase, chitinase, and protease) has been detected. Furthermore, applying biocontrol agents, especially *T. viride*, increased the G% and vigor index of coriander seedlings compared to traditional fungicides. Although the conventional fungicides, especially Amistar 25% SC, showed higher efficacy than the tested biocontrol agents in decreasing the disease incidence and severity, the biocontrol agents significantly alleviated the incidence of damping-off and root rot/wilt severity of infected coriander plants compared with the control. Moreover, applying the biocontrol agents induced coriander resistance to *R. solani* by increasing the resistance-related compounds and antioxidant enzymes leading to high protection of coriander seedlings against *R. solani* and other biotic and abiotic stresses. Thus, these biocontrol agents may be a good substitution for conventional fungicides for the best organic agriculture and ecosystem sustainability.

## Figures and Tables

**Table 1:** The half-maximal inhibitory concentration (IC) values (ppm) for Amistar 25% SC and Rizolex-T 50% WP.

Fungicides	IC	Concentration (ppm)	95% Confidence Interval	
			Lower	Upper
Amistar 25% SC	50	0.64032	0.46491	0.81904
	90	1.23583	1.00941	1.72762
Rizolex-T 50% WP	50	0.76212	0.49435	1.11969
	90	1.35869	1.03802	2.55942

**Table 2:** Effect of different bioagent treatments on damping-off and root rot/wilt caused by *Rhizoctonia solani* *.

Treatments	Pre-Emergence Damping-off	Post-Emergence Damping-off	Root Rot/Wilt	
			DI	DS
Control	54.1 ^a^	38.3 ^a^	25.2 ^a^	15.1 ^a^
*T. viride*	17.8 ^d^	11.3 ^b^	16.1 ^b,c^	7.5 ^c,d^
*P. fluorescence*	19.6 ^c^	12.4 ^b^	14.1 ^c,d^	8.5 ^c^
*B. subtilis*	24.1 ^b^	13.5 ^b^	17.3 ^b^	10.5 ^b^
Amistar 25% SC	11.8 ^f^	7.8 ^c^	11.4 ^e^	4.9 ^e^
Rizolex-T 50% WP	14.3 ^e^	10.3 ^b,c^	12.9 ^d,e^	6.2 ^d,e^

* Values presented as the means of five replicates. Different letters indicate statistically significant differences among treatments, while the same letters signify no significant differences between them according to a Tukey’s honestly significant difference test (*p* < 0.05).

## Data Availability

The authors will freely share the unprocessed raw data that underlies the results of this article.

## References

[1] Wang X.J., Luo Q., Li T., Meng P.H., Pu Y.T., Liu J.X., Xiong A.S. (2022). Origin, evolution, breeding, and omics of Apiaceae: A family of vegetables and medicinal plants. Hortic. Res..

[2] Bhuiyan N.I., Begum J., Sultana M. (2009). Chemical composition of leaf and seed essential oil of *Coriandrum sativum* L. from Bangladesh. Bangladesh J. Pharmacol..

[3] Chahal K.K., Singh R., Kumar A., Bhardwaj U. (2017). Chemical composition and biological activity of *Coriandrum sativum* L.: A review. Indian J. Nat. Prod. Res..

[4] Jabeen Q., Bashir S., Lyoussi B., Gilani A.H. (2009). Coriander fruit exhibits gut modulatory, blood pressure lowering and diuretic activities. J. Ethnopharmacol..

[5] Bhaliya C.M., Jadeja K.B. (2014). Efficacy of different fungicides against *Fusarium solani* causing coriander root rot. Bioscan.

[6] Sneh B., Coautor L.B., Coautor A.O. (1991). Identification of Rhizoctonia Species. No. 581 Sn26i Ej. 1 004809.

[7] Koike S.T., Daugovish O., Martin F.N., Ramon M.L. (2017). Crown and Root Rot Caused by *Rhizoctonia solani* on Cilantro in California. Plant Dis..

[8] Saleh E., El-Samman M., Abu El-Wafa W., Sharaf M., Ahmed F. (2013). Biocontrol of damping-off disease caused by *Rhizoctonia solani* in some medicinal plants using local strain of *Streptomyces pactum*. Aust. J. Basic Appl. Sci..

[9] Nada M., Imarah D., Halaw A. (2014). Efficiency of some silicon sources for controlling damping-off of coriander (*Coriandrum sativum* L.) in Egypt. Egypt. J. Phytopathol..

[10] Senapati M., Tiwari A., Sharma N., Chandra P., Bashyal B.M., Ellur R.K., Krishnan S.G. (2022). *Rhizoctonia solani* Kühn pathophysiology: Status and prospects of sheath blight disease management in rice. Front. Plant Sci..

[11] Balba H. (2007). Review of strobilurin fungicide chemicals. J. Environ. Sci. Health Part B.

[12] Gisi U., Sierotzki H., Cook A., McCaffery A. (2002). Mechanisms influencing the evolution of resistance to Qo inhibitor fungicides. Pest Manag. Sci..

[13] Hefnawy M.A., Eisa O.A., El-Feky N.M. (2012). Impact of the fungicide Rizolix T50% on the antagonistic activity of *Trichoderma harzianum* and *Trichoderma koningii*. Int. J. Sci. Res..

[14] O’Brien P.A. (2017). Biological control of plant diseases. Australas. Plant Pathol..

[15] Collinge D.B., Jensen D.F., Rabiey M., Sarrocco S., Shaw M.W., Shaw R.H. (2022). Biological control of plant diseases—What has been achieved and what is the direction?. Plant Pathol..

[16] Guzmán-Guzmán P., Porras-Troncoso M.D., Olmedo-Monfil V., Herrera-Estrella A. (2019). *Trichoderma* species: Versatile plant symbionts. Phytopathology.

[17] Hariharan G., Rifnas L.M., Prasannath K., Kumar A. (2022). Role of *Trichoderma* spp. in Biocontrol of Plant Diseases. Microbial Biocontrol: Food Security and Post Harvest Management.

[18] Thakur R., Srivastava S., Yadav S. (2023). Multi trait *Pseudomonas* sp. isolated from the rhizosphere of *Bergenia ciliata* acts as a growth-promoting bioinoculant for plants. Front. Sustain. Food Syst..

[19] El Komy M.H., Saleh A.A., Eranthodi A., Molan Y.Y. (2015). Characterization of novel *Trichoderma asperellum* isolates to select effective biocontrol agents against tomato Fusarium wilt. Plant Pathol. J..

[20] Zin N.A., Badaluddin N.A. (2020). Biological functions of *Trichoderma* spp. for agriculture applications. Ann. Agric. Sci..

[21] Dutta P., Deb L., Pandey A.K. (2022). Trichoderma-from lab bench to field application: Looking back over 50 years. Front. Agron..

[22] Harman G.E., Howell C.R., Viterbo A., Chet I., Lorito M. (2004). *Trichoderma* species-opportunistic, avirulent plant symbionts. Nat. Rev. Microbiol..

[23] Vinale F., Sivasithamparam K., Ghisalberti E.L., Marra R., Barbetti M.J., Li H., Woo S.L., Lorito M. (2008). A novel role for *Trichoderma* secondary metabolites in the interactions with plants. Physiol. Mol. Plant Pathol..

[24] Vinale F., Sivasithamparam K., Ghisalberti E.L., Marra R., Woo S.L., Lorito M. (2008). *Trichoderma*-plant-pathogen interactions. Soil Biol. Biochem..

[25] Van Wees S.C.M., Van der Ent S., Pieterse C.M.J. (2008). Plant immune responses triggered by beneficial microbes. Curr. Opin. Plant Biol..

[26] Grover M., Ali S.Z., Sandhya V., Rasul A., Venkateswarlu B. (2011). Role of microorganisms in adaptation of agriculture crops to abiotic stresses. World J. Microbiol. Biotechnol..

[27] Vejan P., Abdullah R., Khadiran T., Ismail S., Nasrulhaq B.A. (2016). Role of plant growth promoting Rhizobacteria in agricultural sustainability—A Review. Molecules.

[28] Mahapatra S., Yadav R., Ramakrishna W. (2022). *Bacillus subtilis* impact on plant growth, soil health, and environment: Dr. Jekyll and Mr. Hyde. J. Appl. Microbiol..

[29] Vanitha S., Ramjegathesh R. (2014). Biocontrol potential of *Pseudomonas fluorescens* against coleus root rot disease. J. Plant Pathol. Microb..

[30] Xu W.F., Ren H.S., OU T., Lei T., Wei J.H., Huang C.S., LI T., Strobel G., Zhou Z.Y., Xie J. (2019). Genomic and functional characterization of the endophytic *Bacillus subtilis* 7PJ-16 strain, a potential biocontrol agent of mulberry fruit sclerotiniose. Microb. Ecol..

[31] Goswami D., Thakker J.N., Dhandhukia P.C. (2016). Portraying mechanics of plant growth promoting rhizobacteria (PGPR): A review. Cogent Food Agric..

[32] Zahra S.T., Tariq M., Abdullah M., Azeem F., Ashraf M.A. (2023). Dominance of Bacillus species in the wheat (*Triticum aestivum* L.) rhizosphere and their plant growth promoting potential under salt stress conditions. PeerJ.

[33] Kubicek C.P., Herrera-Estrella A., Seidl-Seiboth V., Martinez D.A., Druzhinina I.S., Thon M., Zeilinger S., Casas-Flores S., Horwitz B.A., Mukherjee P.K. (2011). Comparative genome sequence analysis underscores mycoparasitism as the ancestral lifestyle of *Trichoderma*. Genome Biol..

[34] Sood M., Kapoor D., Kumar V., Sheteiwy M.S., Ramakrishnan M., Landi M., Araniti F., Sharma A. (2020). *Trichoderma*: The “secrets” of a multitalented biocontrol agent. Plants.

[35] Daguerre Y., Edel-Hermann V., Steinberg C. (2017). Fungal genes and metabolites associated with the biocontrol of soil-borne plant pathogenic fungi. Fungal Metabolites.

[36] Alizadeh M., Vasebi Y., Safaie N. (2020). Microbial antagonists against plant pathogens in Iran: A review. Open Agric..

[37] Loc N.H., Huy N.D., Quang H.T., Lan T.T., Ha T.T.T. (2020). Characterization and antifungal activity of extracellular chitinase from a biocontrol fungus, *Trichoderma asperellum* PQ34. Mycology.

[38] Chen X.Y., Zhang Y.Y., Fu X.C., Li Y., Wang Q. (2016). Isolation and characterization of *Bacillus amyloliquefaciens*, PG12 for the biological control of apple ring rot. Postharvest Biol. Technol..

[39] Velazhahan R., Samiyappan R., Vidhyasekaran P. (1999). Relationship between antagonistic activities of *Pseudomonas fluorescens* strains against *Rhizoctonia solani* and their production of lytic enzymes. J. Plant Dis. Prot..

[40] Meena B., Marimuthu T., Vidhyasekaran P., Velazhahan R. (2001). Biological control of root rot of groundnut with antagonistic *Pseudomonas fluorescens* strains. J. Plant Dis. Prot..

[41] Nagarajkumar M., Bhaskaran R., Velazhahan R. (2004). Involvement of secondary metabolites and extracellular lytic enzymes produced by *Pseudomonas fluorescens* in inhibition of *Rhizoctonia solani*, the rice sheath blight pathogen. Microbiol. Res..

[42] Kell C., Schnider U., Maurhofer M., Voisard C., Laville J., Burger U., Wirthner P., Hass D., Defago G. (1992). Suppression of root diseases by *Pseudomonas fluorescens* CHAO: Importance of the bacterial secondary metabolite 2,4-diacetylphloroglucinol. Mol. Plant-Microbe Interact..

[43] Flaishman M.A., Eyal Z., Zilberstein A., Voisard C., Haas D. (1996). Suppression of *Septoria tritici* blotch and leaf rust of wheat by recombinant cyanide-producing strains of *Pseudomonas putida*. Mol. Plant-Microbe Interact..

[44] Chowdhury S.P., Hartmann A., Gao X., Borriss R. (2015). Biocontrol mechanism by root-associated *Bacillus amyloliquefaciens* FZB42. A review. Front. Microbiol..

[45] Niu D.D., Wang X.J., Wang Y.R., Song X.O., Wang J.S., Guo J.H., Zhao H.W. (2016). *Bacillus cereus* AR156 activates PAMP-triggered immunity and induces a systemic acquired resistance through a NPR1-and SA-dependent signaling pathway. Biochem. Biophys. Res. Commun..

[46] Meng X., Miao Y., Liu Q., Ma L., Guo K., Liu D., Ran W., Shen Q. (2019). TgSWO from *Trichoderma guizhouense* NJAU4742 promotes growth in cucumber plants by modifying the root morphology and the cell wall architecture. Microb. Cell Factories.

[47] Khan M.R., Parveen G. (2018). Supplementing biocontrol agents with botanicals improved growth and yield of coriander (*Coriandrum sativum* L.) infected with *Protomycesmacrosporus* Unger. Curr. Plant Biol..

[48] Cai W.J., Ye T.T., Wang Q., Cai B.D., Feng Y.Q. (2016). A rapid approach to investigate spatiotemporal distribution of phytohormones in rice. Plant Methods.

[49] Glick B.R. (1995). The enhancement of plant growth by free-living bacteria. Can. J. Microbiol..

[50] Leeman M., den Ouden F.M., Van Pelt J.A., Dirkx F.P.M., Steijl H., Bakker P.A.H.M., Schippers B. (1996). Iron availability affects induction of systemic resistance to *Fusarium* wilt of radish by *Pseudomonas fluorescens*. Phytopathology.

[51] Youssef S.A., Tartoura K.A., Abdelraouf G.A. (2016). Evaluation of *Trichoderma harzianum* and *Serratia proteamaculans* effect on disease suppression, stimulation of ROS-scavenging enzymes and improving tomato growth infected by *Rhizoctonia solani*. Biol. Control.

[52] Bagy H.M.K., Hassan E.A., Nafady N.A., Dawood M.F. (2019). Efficacy of arbuscular mycorrhizal fungi and endophytic strain Epicoccum nigrum ASU11 as biocontrol agents against the blackleg disease of potato caused by bacterial strain *Pectobacteriumcarotovora* subsp. *atrosepticum* PHY7. Biol. Control.

[53] Sofy M., Mohamed H., Dawood M., Abu-Elsaoud A., Soliman M. (2021). Integrated usage of *Trichoderma harzianum* and biochar to ameliorate salt stress on spinach plants. Arch. Agron. Soil Sci..

[54] Abdelrhim A.S., Yasser S.A.M., Nehela Y., Atallah O.O., El-Ashmony R.M., Dawood M.F.A. (2021). Silicon dioxide nanoparticles induce innate immune responses and activate antioxidant machinery in wheat against *Rhizoctonia solani*. Plants.

[55] Dawood M.F., Sofy M.R., Mohamed H.I., Sofy A.R., Abdel-Kader H.A. (2023). N-or/and P-deprived *Coccomyxachodatii* SAG 216–2 extracts instigated mercury tolerance of germinated wheat seedlings. Plant Soil.

[56] Dawood M.F., Sofy M.R., Mohamed H.I., Sofy A.R., Abdel-kader H.A. (2022). Hydrogen sulfide modulates salinity stress in common bean plants by maintaining osmolytes and regulating nitric oxide levels and antioxidant enzyme expression. J. Soil Sci. Plant Nutr..

[57] Dawood M.F., Tahjib-Ul-Arif M., Sohag A.A.M., Abdel Latef A.A.H. (2022). Fluoride mitigates aluminum-toxicity in barley: Morpho-physiological responses and biochemical mechanisms. BMC Plant Biol..

[58] Chowdappa P., Kumar S.P.M., Lakshmi M.J., Upreti K.K. (2013). Growth stimulation and induction of systemic resistance in tomato against early and late blight by *Bacillus subtilis* OTPB1 or *Trichoderma harzianum* OTPB3. Biol. Control.

[59] Kumar V., Parkhi V., Kenerley C.M., Rathore K.S. (2009). Defense-related gene expression and enzyme activities in transgenic cotton plants expressing an endochitinase gene from *Trichoderma virens* in response to interaction with *Rhizoctonia solani*. Planta.

[60] Nawrocka J., Małolepsza U., Szymczak K., Szczech M. (2018). Involvement of metabolic components, volatile compounds, PR proteins, and mechanical strengthening in multilayer protection of cucumber plants against *Rhizoctonia solani* activated by *Trichoderma atroviride* TRS25. Protoplasma.

[61] Chandrasekaran M., Chun S.C. (2016). Expression of PR-protein genes and induction of defense-related enzymes by *Bacillus subtilis* CBR05 in tomato (*Solanum lycopersicum*) plants challenged with *Erwinia carotovora* subsp. Carotovora. Biosci. Biotechnol. Biochem..

[62] Sallam N.M., AbdElfatah H.A.S., Dawood M.F., Hassan E.A., Mohamed M.S., Khalil Bagy H.M. (2021). Physiological and histopathological assessments of the susceptibility of different tomato (*Solanum lycopersicum*) cultivars to early blight disease. Eur. J. Plant Pathol..

[63] Zhao H., Xiaobing W., Wei W. (2021). *Bacillus amyloliquefaciens* SN16-1-induced resistance system of the tomato against *Rhizoctonia solani*. Pathogens.

[64] Abdelrhim A.S., Dawood M.F., Galal A.A. (2022). Hydrogen peroxide-mixed compounds and/or microwave radiation as alternative control means against onion seed associated pathogens, *Aspergillus niger* and *Fusarium oxysporum*. J. Plant Pathol..

[65] Manganiello G., Sacco A., Ercolano M.R., Vinale F., Lanzuise S., Pascale A., Napolitano M., Lombardi N., Lorito M., Woo S.L. (2018). Modulation of tomato response to *Rhizoctonia solani* by *Trichoderma harzianum* and its secondary metabolite harzianic acid. Front. Microbiol..

[66] Zhang Y., Zhuang W.Y. (2020). *Trichoderma brevicrassum* strain TC967 with capacities of diminishing cucumber disease caused by *Rhizoctonia solani* and promoting plant growth. Biol. Control.

[67] Zhang J.L., Tang W.L., Huang Q.R., Li Y.Z., Wei M.L., Jiang L.L., Liu C., Yu X., Zhu H.W., Chen G.Z. (2021). *Trichoderma*: A treasure house of structurally diverse secondary metabolites with medicinal importance. Front. Microbiol..

[68] Abbas A., Mubeen M., Zheng H., Sohail M.A., Shakeel Q., Solanki M.K., Iftikhar Y., Sharma S., Kashyap B.K., Hussain S. (2022). *Trichoderma* spp. genes involved in the biocontrol activity against *Rhizoctonia solani*. Front. Microbiol..

[69] Jaroszuk-Ściseł J., Tyśkiewicz R., Nowak A., Ozimek E., Majewska M., Hanaka A., Tyśkiewicz K., Pawlik A., Janusz G. (2019). Phytohormones (auxin, gibberellin) and ACC deaminase in vitro synthesized by the mycoparasitic *Trichoderma* DEMTkZ3A0 strain and changes in the level of auxin and plant resistance markers in wheat seedlings inoculated with this strain conidia. Int. J. Mol. Sci..

[70] Jain S., Vaishnav A., Kumari S., Varma A., Tuteja N., Choudhary D.K. (2017). Chitinolytic *Bacillus*-mediated induction of jasmonic acid and defense-related proteins in soybean (*Glycine max*, L. Merrill) plant against *Rhizoctonia solani*, and *Fusarium oxysporum*. J. Plant Growth Regul..

[71] Koley P., Brahmachari S., Saha A., Deb C., Mondal M., Das N., Das A., Lahiri S., Das M., Thakur M. (2022). Phytohormone priming of tomato plants evoke differential behavior in *Rhizoctonia solani* during infection, with salicylate priming imparting greater tolerance than jasmonate. Front. Plant Sci..

[72] Niu D.D., Liu H.X., Jiang C.H., Wang Y.P., Wang Q.Y., Jin H.L., Guo J.H. (2011). The plant growth–promoting rhizobacterium *Bacillus cereus* AR156 induces systemic resistance in *Arabidopsis thaliana* by simultaneously activating salicylate- and jasmonate/ethylene-dependent signaling pathways. Mol. Plant-Microbe Interact..

[73] Chowdhury S.P., Uhl J., Grosch R., Alqueres S., Pittroff S., Dietel K., Schmitt-Kopplin P., Borriss R., Hartmann A. (2015). Cyclic lipopeptides of *Bacillus amyloliquefaciens* subsp. *plantarum* colonizing the lettuce rhizosphere enhance plant defense responses toward the bottom rot pathogen *Rhizoctonia solani*. Mol. Plant Microbe Interact..

[74] Poveda J., Barquero M., Gonzalez-Andres F. (2020). Insight into the microbiological control strategies against *Botrytis cinerea* using systemic plant resistance activation. Agronomy.

[75] Maurhofer M., Reimmann C., Schmidli-sacherer P., Heeb S., Haas D., Defago G. (1998). Salicylic acid biosynthetic genes expressed in *Pseudomonas fluorescens* strain P3 improve the induction of system resistance in tobacco against tobacco necrosis virus. Phytopathology.

[76] Gaffney T., Friedrich L., Vernooji B., Negrotto D., Nye G., Uknes S., Ward E., Kessman H., Ryals J. (1993). Requirement of salicylic acid for induction of systemic acquired resistance. Science.

[77] Lessy M.N.A., Abdelrhim A.S., Abdel-Aziz N.A., Saleh O.I., Abdel-Latif M.R. (2022). Survey on incidence of damping-off and root rot/wilt diseases of coriander (*Coriandrun sativum* L.) in Minia Governorate. Minia J. Agric. Res. Dev..

[78] Ireland K.B., Weir B.S., Phantavong S., Phitsanoukane P., Vongvichid K., Vilavong S., Tesoriero L.A., Burgess L.W. (2015). First report of *Rhizoctonia solani* anastomosis group AG-4 HG-I in the Lao PDR. Australas. Plant Dis. Notes.

[79] Al-Fadhal F.A., AL-Abedy A.N., Alkhafije D.A. (2019). Isolation and molecular identification of *Rhizoctonia solani* and *Fusarium solani* isolated from cucumber (*Cucumis sativus* L.) and their control feasibility by *Pseudomonas fluorescens* and *Bacillus subtilis*. Egypt. J. Biol. Pest Control.

[80] Beale R.E., Phillion D.P., Headrick J.M., O’Reilly P., Cox J. (1998). MON65500: A unique fungicide for the control of take-all in wheat. Proceedings of the 1986 British Crop Protection Conference—Pestsand Diseases.

[81] Prentice R.L. (1976). A generalization of the probit and logit methods for dose response curves. Biometrics.

[82] Hassan G.M., El-Feky Z.A., Hemada N.F., Sayed M.A. (2015). Isolation and molecular characterization of Egyptian *Trichoderma* and assessment of their antagonistic potential against Rhizoctonia solani. J. Microbio. Biotech. Food Sci..

[83] Perveen K., Bokhari N.A. (2012). Antagonistic activity of *Trichoderma harzianum* and *Trichoderma viride* isolated from soil of date palm field against *Fusarium oxysporum*. Afr. J. Microbiol. Res..

[84] Singh R., Singh B.K., Upadhyay R.S., Rai B., Lee Y.S. (2002). Biological control of Fusarium wilt disease of pigeon pea. Plant Pathol. J..

[85] Sukalpa D., Abdul Wadud M.D., Atiqur R.K. (2021). Functional evaluation of culture filtrates of *Bacillus subtilis* and *Pseudomonas fluorescens* on the mortality and hatching of *Meloidogyne javanica*. Saudi J. Biol. Sci..

[86] Mahesha H.S., Ravichandra N.G., Rao M.S., Narasegowda N.C. (2017). Bio-efficacy of different strains of *Bacillus spp*. against *Meloidogyne incognita* under in vitro. Int. J. Curr. Microbiol. Appl. Sci..

[87] Sela S., Schickler H., Chet I., Spiegel Y. (1998). Purification and characterization of *Bacillus cereus* collagenolytic/proteolytic enzyme and its effect on *Meloidogyne javanica* cuticular proteins. Eur. J. Plant Pathol..

[88] Miller G.L. (1959). Use of dinitro salicylic acid reagent for determination of reducing sugar. Anal. Chem..

[89] Mathivanan N., Kabilan V., Murugesan K. (1998). Purification, characterization, and antifungal activity of chitinase from *Fusarium chlamydosporum*, a mycoparasite to groundnut rust, *Puccinia arachidis*. Can. J. Microbiol..

[90] Melo I.S., Faull J.L., Graeme-Cook K.A. (1997). Relationship between in vitro cellulase production of UV-induced mutants of *Trichoderma harzianumand* their bean rhizosphere competence. Mycol. Res..

[91] Lowry O.H., Rosebrough N.J., Farr A.L., Randall R.J. (1951). Protein measurement with the Folin phenol reagent. J. Biol. Chem..

[92] Zhu X., Wang Y., Wang X., Wang W. (2022). Exogenous regulators enhance the yield and stress resistance of chlamydospores of the biocontrol agent *Trichoderma harzianum* T4. J. Fungi.

[93] Vidhyasekaran P., Rabindran R., Muthamilan M., Nayar K., Rajappan K., Subramanian N., Vasumathi K. (1997). Development of powder formulation of *Pseudomonas fluorescens* for control of rice blast. Plant Pathol..

[94] Biyyani S., Vijaya G.A., Subhash R.R., Triveni S., Nissipaul M. (2017). Study the efficacy of against sheath blight in rice by *Rhizoctonia solani*. Int. J. Curr. Microbiol. App. Sci..

[95] Abdul-Baki A.A., Anderson J.D. (1973). Vigour determination of soybean seed by multiple criteria. Crop Sci..

[96] Lichtenthaler H.K. (1987). Chlorophylls and carotenoids: Pigments of photosynthetic biomembranes. Methods Enzymol..

[97] Mukherjee S.P., Choudhuri M.A. (1983). Implications of water stress-induced changes in the levels of endogenous ascorbic acid and hydrogen peroxide in *Vigna* seedlings. Physiol. Plant..

[98] Madhava Rao K.V., Sresty T.V.S. (2000). Antioxidative parameters in the seedlings of pigeon pea (*Cajanuscajan* (L.) *Millspaugh*) in response to Zn and Ni stresses. Plant Sci..

[99] Warrier R.R., Paul M., Vineetha M.V. (2013). Estimation of salicylic acid in Eucalyptus leaves using spectrophotometric methods. Genet. Plant Physiol..

[100] Kofalvi S.A., Nassuth A. (1995). Influence of wheat streak mosaic virus infection on phenylpropanoid metabolism and the accumulation of phenolics and lignin in wheat. Physiol. Mol. Plant Pathol..

[101] Havir E.A., Hanson K.R. (2002). L-Phenylalanine ammonia-lyase (maize and potato). Evidence that the enzyme is composed of four subunits. Biochemistry.

[102] Kumar K.B., Khan P.A. (1982). Peroxidase and polyphenol oxidase in excised ragi (*Eleusine corocana* cv. PR 202) leaves during senescence. Indian J. Exp. Biol..

[103] Misra H.P., Fridovich I. (1972). The role of superoxide anion in the autoxidation of epinephrine and a simple assay for superoxide dismutase. J. Biol. Chem..

[104] Noctor G., Mhamdi A., Foyer C.H. (2016). Oxidative stress and antioxidative systems: Recipes for successful data collection and interpretation. Plant. Cell Environ..

[105] Silva E.N., Silveira J.A.G., Aragão R.M., Vieira C.F., Carvalho F.E.L. (2018). Photosynthesis impairment and oxidative stress in Jatropha curcas exposed to drought are partially dependent on decreased catalase activity. Acta Physiol. Plant..

[106] Tatiana Z., Yamashita K., Matsumoto H. (1999). Iron deficiency induced changes in ascorbate content and enzyme activities related to ascorbate metabolism in cucumber root. Plant Cell Physiol..

